# *H.pylori *associated with iron deficiency anemia even in celiac disease patients; strongly evidence based but weakly reflected in practice

**Published:** 2015

**Authors:** Mohammad Rostami-Nejad, David Aldulaimi, Helen Livett, Kamran Rostami

**Affiliations:** 1 *Gastroenterology and Liver Diseases Research Center, Research Institute for Gastroenterology and Liver Diseases, Shahid Beheshti University of Medical Sciences, Tehran, Iran*; 2 *Department of Gastroenterology, Alexandra Hospital, Worcestershire, B98 7UB, United Kingdom*

**Keywords:** *Helicobacter pylori*, Iron deficiency anaemia, Coeliac disease

## Abstract

Inflammation can lead to malabsorption of important micronutrients such as iron. Malabsorption and nutritional deficiency can be caused by a variety of pathological and environmental factors causing a range of other symptoms commonly caused by both *H. pylori* infection and coeliac disease (CD). National guidelines suggest the routine taking of duodenal biopsies to exclude CD when investigating patients for iron deficiency anemia (IDA). Studies suggest that in absence of positive antibodies, IDA is rarely caused by CD. Recent British Society of Gastroenterology guidelines discourage the routine duodenal biopsies in low risk cases but despite this guidance, taking duodenal biopsies for IDA is a common practice. Many studies have reported that *H. pylori* infection is associated with IDA even in patients with CD. In countries with low *H. pylori* prevalence we still detect more *H. pylori* than CD standing behind IDA. Despite the strong association between IDA and *H. pylori*, taking biopsies to diagnose *H. pylori* infection is not usually a routine part of the diagnostic workup to identify the etiology of IDA. In this review we will discuss the impact of *H. pylori* in IDA and highlight the possible gaps in identifying the IDA etiology.

## Introduction

 Iron deficiency anaemia (IDA) is a common cause of referral to gastroenterologists (4%-13% of referrals) (1) and both coeliac disease (CD) and *H pylori* are associated with IDA. A significant number of patients with IDA (5-10%) present with idiopathic form of IDA with no clear aetiology established, even after extensive examination (2, 3) (Table 1). The effect of *H. pylori *infection on iron absorption is likely to be multifactorial. Several mechanisms may lead to a decrease in iron absorption including hypochlorhydria and decreased ascorbic acid secondary to chronic gastritis (4-8). Another potentially important mechanism is increased hepcidin production from hepatocytes in response to IL-6 production, secondary to *H. pylori *infection*, *IBD, CD or other inflammatory conditions, leading to reduced iron absorption (9). 

A microscopic enteropathy, with an increased numbers of intestinal intraepithelial lymphocytes (IELs), might be associated with IDA. Increased IELs has been reported in disorders such as *H. pylori* infection, CD, giardia infection, IgA deficiency, and Crohn’s disease (10-12). 

**Table 1:** Frequency investigated etiology of iron deficiency anaemia

**Etiology **	**Frequency (%)**
Colonic carcinoma	5-10
Gastric carcinoma	5
Benign gastric ulceration	5
Angiodysplasia	5
Coeliac disease	4-6
Excessive blood loss during menstruation	20-30
Gastric antral vascular ectasia	1-2
NSAIDs related	10-15

Several studies have reported that numbers of intraepithelial lymphocytes in the duodenal mucosa are more likely increased in patients with *H pylori *infection. This subtle small bowel inflammation might be implicated in reduced iron absorption and potentially treated by the *H. pylori *eradication. 

*H. pylori *infection and CD can both be present. Although epidemiological investigations have not confirmed an association between gastritis and CD (13-16), other studies have reported a *H. pylori *related lymphocytic gastritis and anaemia in patients with CD (17). 

Taking duodenal biopsies in every patient with negative coeliac serology presenting with IDA may not be indicated. In this review article we discuss some weaknesses in current policy assessing patients with IDA. 

## Discussion

**Figure 1:**
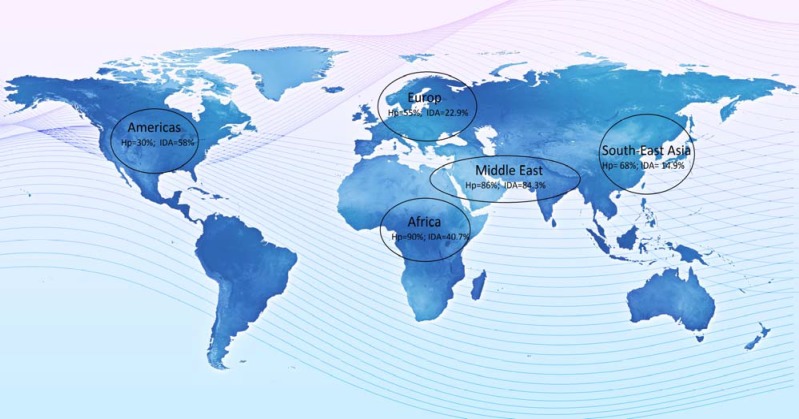
Global map of *H. pylori* (Hp) infection and Iron Deficiency Anemiea (IDA).

Currently, many studies demonstrate a relationship between IDA and CD and *H. pylori* infection. A number of studies have suggested that the frequency of *H. pylori* is high in different parts of the world (24). Around one-third of adults in North American and north European residents are still infected. The prevalence of *H. pylori* infection is often more than 50% in South and Eastern Europe, South America, and Asia (figure 1). In 2005, Hershko et al. prospectively studied 150 IDA patients to confirm the role of *H. pylori *and CD in refractory or unexplained IDA (18). In this study, Hershko et al. aimed to confirm the role of H pylori and CD in refractory or unexplained IDA. The authors reported that H. pylori infection was found in 19% of patients and CD was identified in 15% adult patients. According to this study, H. pylori is a major etiological factor for refractory IDA with a higher association rate compared to CD (table 2). 

Failing to test for H. pylori infection could lead to a failure to identify a treatable cause of anaemia and could lead to additional and potentially unnecessary investigations.

**Table 2:** Other controverted common etiology of iron deficiency anaemia, less frequently investigated

H pylori infection
Chronic kidney disease
Gastrointestinal or systemic
Inflammations/autoimmune disorders
Non-coeliac gluten sensitivity

The study suggests that routine testing for *H. pylori* in IDA would be appropriate. Cuoco et al. examined the relationship between *H. pylori* and IDA in 362 patients with CD (19). *H. pylori *infection was present in 21% (77) of cases. Among the remaining *H. pylori*-negative subjects, 28% (81 cases) showed anaemia (*P*< 0.001). The results of this study showed a significant association between *H. pylori *infection and IDA in patients with CD. This study confirms that *H. pylori* might be another major etiology of IDA even in patients with CD. It might be entirely appropriate and cost effective to incorporate *H. pylori* investigations in patients with IDA and those patients who have a diagnosis of CD with persistent anaemia in addition to a gluten free diet. The evidence behind the *H. pylori* associated IDA in these studies (16-18) strongly suggest that the order of investigation in clinical practice needs to be revised, with the introduction of routine early testing for *H. pylori *infection into standard practice. 

*H.pylori* and CD frequently are found in association with refractory IDA (20). Refractory IDA may be due to clinically asymptomatic *H. pylori* infection. Treating CD and eradication of *H. pylori *with additional iron therapy may assist with the correction of anaemia (21). The association between CD and *H. pylori* is controversial. However in the presence of IDA the association between these 2 conditions has become more significant according to Demir et al (22). 

In countries where little meat is in the diet, IDA is more prevalent than in North Americas and Europe.

**Figure 2:**
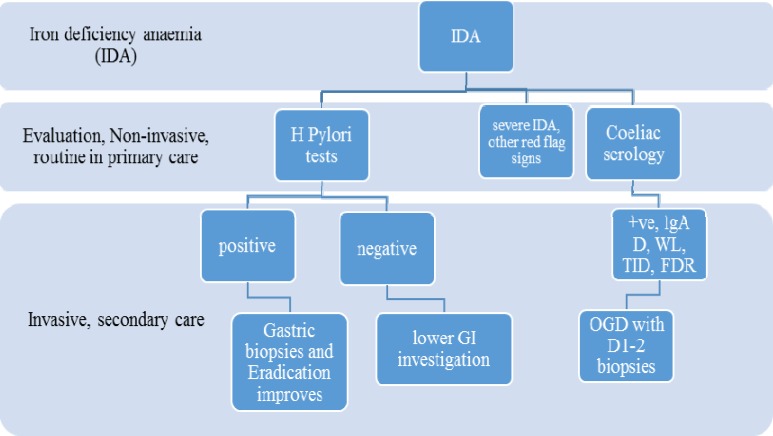
Proposed Algorithm

IDA: Iron deficiency anemia; IgA D; IgA deficiency, WL; weight loss, TID; type I diabetes, FDR; first degree relative of coeliac patients

The result of their study showed that 42% of patients with CD were infected with H. pylori and 7 (47%) of them had iron deficiency anemia. They suggested that CD itself plays a major role in the development of IDA. Contrary to the majority of studies Simondi et al demonstrated that the frequency of H. pylori infection was not significantly different in CD patients with or without IDA (23).

## Conclusion

*H. pylori* infection has a greater prevalence in North America, south/east Europe Asian and Middle Eastern countries. The global prevalence of CD has less variation, with exception of those countries that have traditionally a lower dietary gluten intake. A proportion of patients with IDA might have anaemia secondary to *H.pylori *infection, especially in countries where *H. pylori* is very common. There are many studies that report* H. pylori* infection as a strong etiological factor for IDA, even among patients with CD. The importance of *H. pylori *infection as a common and readily reversible cause of IDA is not reflected appropriately in current guidelines and routine clinical practice. Investigation and eradication of *H.pylori* should be incorporated into IDA diagnostic workup, especially in populations where infection is endemic. Achlorhydric gastric atrophy is a common evidence based etiology for IDA. Therefore gastric biopsies should be taken in patients with no other explanation for anaemia (25) (Figure 2).
